# Circulating LIGHT (TNFSF14) and Interleukin-18 Levels in Sepsis-Induced Multi-Organ Injuries

**DOI:** 10.3390/biomedicines10020264

**Published:** 2022-01-25

**Authors:** Hui-Qi Qu, James Snyder, John Connolly, Joseph Glessner, Charlly Kao, Patrick Sleiman, Hakon Hakonarson

**Affiliations:** 1 The Center for Applied Genomics, Children’s Hospital of Philadelphia, Philadelphia, PA 19104, USA; quh@email.chop.edu (H.-Q.Q.); snyderj3@email.chop.edu (J.S.); connollyJ1@email.chop.edu (J.C.); glessner@email.chop.edu (J.G.); kaoc@email.chop.edu (C.K.); sleimanp@email.chop.edu (P.S.); 2 Department of Pediatrics, The Perelman School of Medicine, University of Pennsylvania, Philadelphia, PA 19104, USA; 3 Division of Human Genetics, Children’s Hospital of Philadelphia, Philadelphia, PA 19104, USA; 4 Division of Pulmonary Medicine, Children’s Hospital of Philadelphia, Philadelphia, PA 19104, USA

**Keywords:** acute hypoxic respiratory failure, acute kidney injury, acute respiratory distress syndrome, interleukin-18, LIGHT, sepsis, viral infections

## Abstract

The novel therapeutic target cytokine LIGHT (TNFSF14) was recently shown to play a major role in COVID-19-induced acute respiratory distress syndrome (ARDS). This study aims to investigate the associations of plasma LIGHT and another potentially targetable cytokine, interleukin-18 (IL-18), with ARDS, acute hypoxic respiratory failure (AHRF), or acute kidney injury (AKI), caused by non-COVID-19 viral or bacterial sepsis. A total of 280 subjects diagnosed with sepsis, including 91 cases with sepsis triggered by viral infections, were investigated in this cohort study. Day 0 plasma LIGHT and IL-18, as well as 59 other biomarkers (cytokines, chemokines, and acute-phase reactants) were measured by sensitive bead immunoassay and associated with symptom severity. We observed significantly increased LIGHT level in both bacterial sepsis patients (*p* = 1.80 × 10^−5^) and patients with sepsis from viral infections (*p* = 1.78 × 10^−3^). In bacterial sepsis, increased LIGHT level was associated with ARDS, AKI, and higher Apache III scores, findings also supported by correlations of LIGHT with other biomarkers of organ failure. IL-18 levels were highly variable across individuals and consistently correlated with Apache III scores, mortality, and AKI in both bacterial and viral sepsis. There was no correlation between LIGHT and IL-18. For the first time, we demonstrate independent effects of LIGHT and IL-18 in septic organ failure. The association of plasma LIGHT with AHRF suggests that targeting the pathway warrants exploration, and ongoing trials may soon elucidate whether this is beneficial. Given the large variance of plasma IL-18 among septic subjects, targeting this pathway requires precise application.

## 1. Introduction

The cytokine LIGHT (CD258), also known as tumor necrosis factor superfamily member 14 (TNFSF14), is a secreted protein of the TNF superfamily, recognized by the herpesvirus entry mediator (HVEM, also known as TNFRSF14), the lymphotoxin B receptor (LTβR, also known as TNFRSF3), and decoy receptor DcR3 (also known as TNFRSF6B) [1]. LIGHT exhibits inducible expression and competes with herpes virus glycoprotein D for binding to HVEM on T lymphocytes [2]. LIGHT is a ligand for TNFRSF14, which is a member of the tumor necrosis factor receptor superfamily, also known as HVEM ligand (HVEML). This protein functions as a costimulatory factor for the activation of lymphoid cells aimed at opposing infection by herpesvirus [2]. It additionally stimulates the proliferation of T cells and triggers the apoptosis of various tumor cells [3]. In addition to LIGHT, interleukin-18 (IL-18) has been repeatedly highlighted as a major player in sepsis [4,5]. As the interferon-γ-inducing factor and a central inflammatory mediator, IL-18 has been demonstrated to play important roles in septic shock, organ failure, and sepsis mortality, suggesting a potential therapeutic target in sepsis [6,7,8]. Treatment with anti-IL-18 neutralizing antibodies has been shown to have significantly protective effects in a mouse model of sepsis [9].

Inflammatory reactions and immune responses are known to be dysregulated and compromised in patients with sepsis, the syndrome of infection complicated by organ injuries [10,11]. In this regard, acute respiratory distress syndrome (ARDS) and acute kidney injury (AKI) are major complications of severe infections [12,13], while the mechanisms of immune dysfunction causing these complications remain unknown. Sepsis typically manifests as an uncontrolled systemic inflammatory process, involving the microvasculature of multiple organ systems and manifesting as organ injury in one or multiple organs, including ARDS, AKI, disseminated intravascular coagulation (DIC) [14], or delirium [15,16]. Liver, heart, and brain failures are also relatively common manifestations.

LIGHT has recently come into the spotlight as a potent proinflammatory mediator and has been suggested as an important therapeutic target for immune regulation due to its central role in the function of activated T cells [17,18,19]. A previous study showed that soluble LIGHT induces proinflammatory changes in endothelial cells under systemic inflammatory activation [20]. The potential contribution of LIGHT to both inflammation and tissue fibrosis identifies it as a compelling potential contributor to ARDS and particularly fibroproliferative ARDS, which has very poor clinical outcomes. A recent study suggested an important role of LIGHT/HVEM expression in experimental lung injury in mice [21]. Nonetheless, it is worth attention that LIGHT may act as a double-edged sword. Due to its role in the innate immune response and inflammation recovery, *Tnfsf14*(−/−) mice develop more severe disease pathogenesis of intestinal inflammation [22]. For this reason, our working hypothesis is that LIGHT plays its detrimental effects in sepsis in a binomial mode, i.e., only when its level is higher than a particular threshold. Among the LIGHT receptors, both HVEM and LTβR may drive the inflammatory response initiated by LIGHT [23]. In contrast, the decoy receptor DcR3 is able to neutralize the function of LIGHT [24]. Through this modulatory effect, our previous study suggested that loss of function of the DcR3 gene may be involved in the genetic risk of inflammatory bowel disease (IBD) [25]. A high level of LIGHT beyond a threshold may also exceed the limit of DcR3 modulation.

Among other inflammatory biomarkers measured in this study, IL-18 is another pro-inflammatory LIGHT-like cytokine known to activate and regulate both innate and adaptive immunity, which we have an opportunity to modulate with an anti-IL18 neutralizing mAb. Its dysregulation has been shown to result in autoinflammatory diseases [26,27]. While IL-18 neutralizing mAb has been suggested for the treatment of various autoimmune and autoinflammatory diseases by experimental studies and clinical trials [28,29], its role in sepsis is less clear.

We sought to investigate the potential role of LIGHT and IL18 in bacterial- and viral-induced sepsis unrelated to COVID-19, and specifically the proteins’ associations with ARDS, acute hypoxic respiratory failure, and AKI as major sepsis complications in 280 patients with sepsis. Of those, 189 had either culture-proven or presumed bacterial sepsis and 91 had PCR-diagnosed viral sepsis that resulted in hospitalization and admission to the intensive care unit (ICU). To contextualize the inflammatory cytokine milieu given the relative novelty of LIGHT, we also measured 59 inflammation or inflammasome biomarkers. This study highlights the significant roles of LIGHT and IL-18 in the severity of sepsis, and, for the first time, we demonstrate a key damaging role of LIGHT in patients with sepsis complicated by ARDS or multi-organ failure.

## 2. Materials and Methods

### 2.1. Subjects

This is a nested study with cases drawn from a molecular cohort study. Consecutive subjects (*n* = 191) with sepsis enrolled to the Molecular Epidemiology of Sepsis in the ICU (MESSI) cohort study [30,31] between 2016 and 2017 were included in this plasma investigation. As only two subjects during this time had viral sepsis, to enrich our population for viral sepsis given the reports of LIGHT associating with ARDS due to COVID-19, we broadened our time period from 2014 and 2019 to capture all subjects with viral sepsis during this time, which added 89 subjects. As a result, 189 (67.5%) cases diagnosed with bacterial sepsis and 91 (32.5%) cases diagnosed with PCR-confirmed viral sepsis were included in this study (Table 1). Each cohort was compared respectively with 22 controls from a random deidentified dataset of healthy (population based) subjects within comparable age, sex, and ethnic background to the sepsis cases. As previously published [30,31], subjects were eligible for MESSI if, at the time of admission to the intensive care unit, they had strongly suspected infection and evidence of acute organ failure consistent with Sepsis-3; prior to 2016, we used the consensus definition for “severe sepsis” described in Sepsis-2 [32]. We obtained cold residual plasma on day 0 from the blood collected clinically in the emergency room or on admission to the intensive care unit for subjects transferred from the floor. The clinical protocol is to spin citrated vacutainers upon receipt and to keep the plasma at 4 °C after centrifuging; our research team collected the plasma 12–36 h after centrifuging, and then aliquoted and froze the plasma at −80 °C until assay.

### 2.2. Phenotype Determination

Subjects were enrolled prior to culture positivity, and then all microbiologic data from 7 days prior to and 7 days after day 0 (ICU admission) were reviewed by physician investigators to classify primary and, if present, secondary body site of infection and to record the pathogens identified. The classification of ARDS required meeting oxygen and radiographic Berlin definition criteria on the same calendar day while receiving invasive ventilation [33]. We defined acute hypoxic respiratory failure (AHRF) as the patient manifesting hypoxia with arterial oxygen tension relative to fraction of inspired oxygen (PaO_2_:FiO_2_ or PF ratio) ≤300 with or without mechanical ventilation. The determination of AKI was made in patients without preexisting end stage renal disease, according to serum creatinine, using the Kidney Disease Improving Global Outcomes (KDIGO) guidelines [34]. To adjust for severity of illness at admission, we calculated the Acute Physiology and Chronic Illness III (APACHE III) score [35].

### 2.3. Measurement of Circulating Cytokines

We measured plasma LIGHT and IL-18, as well as an additional 59 biomarkers (cytokines, chemokines, and acute-phase reactants), using the Quanterix high-sensitivity single-molecular array technology (Myriad RBM Simoa™ Services, Austin, TX, USA), with LIGHT as a customized addition to the Human Inflammation MAP^®^ v. 1.1. The list of biomarkers measured is shown in Table S1.

### 2.4. Data Analysis

The plasma levels of the measured biomarkers were normalized by natural log transformation for further analysis, due to the large dynamic ranges of the biomarkers. Elevated LIGHT level was defined as >2 standard deviations (SDs) above the mean in reference controls. Statistical analyses, including independent-samples *t*-test, one-way ANOVA, bivariate correlations, and nonparametric chi-square test, were conducted using the IBM SPSS Statistics Version 23 software. Partial correlation was performed to control the effects of covariates. The *p*-values < 0.001 are presented in scientific notation.

## 3. Results

### 3.1. Sepsis Population Characteristics

The study population included 280 subjects with sepsis, of whom 189 had sepsis of primarily bacterial origin and 91 had PCR-detected viral infection. Characteristics of the population are detailed in Table 1. Lung was the most common primary site of infection, accounting for 55% of sepsis, and 24% manifested bacteremia. For 5% of subjects, the infectious source remained unclear.

Among subjects with viral sepsis, 92% were pulmonary sepsis, and influenza was the most common pathogen (62%) with additional viruses detected including respiratory syncytial virus, human metapneumovirus, adenovirus, non-SARS coronavirus, parainfluenza, rhinovirus/enterovirus, cytomegalovirus, and herpes simplex virus. Among the bacterial sepsis patients, 35% of culture proven cases were Gram-positive bacteria (most commonly *Streptococcus*, *Staphylococcus*, *Enterococcus*, and Gram-positive rod) and 55% were due to Gram-negative bacteria (most commonly *Escherichia coli*, *Pseudomonas aeruginosa*, *Klebsiella pneumoniae*, *Acinetobacter baumannii*, and Gram-negative rod).

Pulmonary sepsis was strongly associated with AHRF (odds ratio, OR 3.29, 95% CI 1.98–5.47, *p* < 0.001) and ARDS (OR 2.32, 95% CI 1.33–3.73, *p* = 0.002). Viral sepsis was more likely to develop AHRF compared to nonviral sepsis (OR 1.96, 95% CI 1.18–3.24, *p* = 0.009) but was not more likely to develop ARDS. Patients developed AKI, with similar rates between viral and nonviral sepsis, and 37% died by day 30.

### 3.2. Increased Plasma LIGHT Levels in Patients with Sepsis 

Increased LIGHT levels were observed in the sepsis cohort of 280 patients overall, compared to control levels from random adult samples, and this was attributed to increased LIGHT levels in both the bacterial and the viral subsets of sepsis (independent *t*-test, Table 2). LIGHT levels were found to correlate inversely with age (*r* = −0.120, *p* = 0.045), i.e., older age correlated with lower levels of LIGHT, whereas sex, race, and BMI did not show correlation with LIGHT levels. Patients with viral sepsis showed a trend of having lower LIGHT levels than patients with bacterial sepsis, albeit not statistically different (*p =* 0.060). In addition, as shown in Table S3, several other biomarkers demonstrated association with organ failure in the overall sepsis group, including both bacterial and viral sepsis.

To examine the cutoff of abnormal LIGHT in an unbiased way, we ran the association test scan with all 189 sepsis cases against 22 controls. Due to potential batch effects between the bacterial sepsis cohort and the viral sepsis cohort, only the bacterial sepsis cohort was scanned. As shown by the results (Table S2), the cutoff of Ln (LIGHT) 4.875 had the lowest false positive rate (1/22) in controls. A total of 138 cases (73%) had a low LIGHT level by this definition. Additionally, we did an association scan for the best cutoff of LIGHT by its association with APACHE III scores. As shown by the results (Table S2), the cutoff 4.913 (142 cases, 75%) had the most statistical significance for the association between LIGHT and APACHE III (*p* = 5.66 × 10^−5^). As both cutoffs 4.875 and 4.913 are close to 5.043, i.e., two standard deviations of the reference controls for Ln (LIGHT), we defined abnormal LIGHT level as Ln (LIGHT) > 5.043. A total of 40 (21.2%) bacterial sepsis cases had elevated LIGHT by this definition.

In the 91 patients with viral sepsis, 12 (13.2%) cases had increased LIGHT. In the 189 bacterial sepsis cases, 40 (21.2%) cases had increased LIGHT, and 1/22 (4.5%) controls had elevated LIGHT. Overall, binary increased LIGHT was associated with AHRF with *r* = 0.123, *p* = 0.043, and a trend toward elevated Apache III score with *r* = 0.114, *p =* 0.060, also associated with age. However, this analysis was underpowered.

In the viral sepsis cases, the Apache III scores were lower compared to bacterial sepsis, fewer patients had elevated LIGHT, and correlation of LIGHT with Apache III score, ARDS, AHRF, AKI, and mortality was not significant. Borderline significance was seen between increased length of ICU/hospital stay (LOS) and elevated LIGHT (*r* = 0.196, *p* = 0.063). Stratified by hospital mortality, the inverse correlation was seen with survival patients (*r* = 0.297, *p* = 0.023), i.e., extended length of hospitalization in survival patients with elevated LIGHT.

In contrast, in the bacterial sepsis cases, elevated LIGHT was correlated with Apache III, with *r* = 0.172, *p* = 0.020, associated with ARDS, with OR (95% CI) = 2.180 (1.077, 4.414), *p* = 0.028, associated with AHRF, with OR (95% CI) = 2.037 (1.011, 4.106), *p* = 0.044, and associated with AKI, with OR (95% CI) = 2.179 (0.991, 4.791), *p* = 0.049. In addition, we observed an inverse correlation between LIGHT concentrations and LOS (β = −0.153, *p* = 0.039). Stratified by hospital mortality, the inverse correlation was seen in patients with hospital mortality, i.e., the higher LIGHT concentration was associated with sooner death (β = −0.235, *p* = 0.040).

Taken together, these results suggest that LIGHT may be a stronger pathogenic driver in bacterial sepsis than in viral sepsis; given that both Apache III scores and mortality rates were higher in the bacterial sepsis, elevated Apache III scores could be considered an approach to identify patients that may have the most benefit from LIGHT neutralizing mAb therapy.

### 3.3. Variable Plasma IL-18 Levels in Patients with Sepsis 

**Figure 1:**
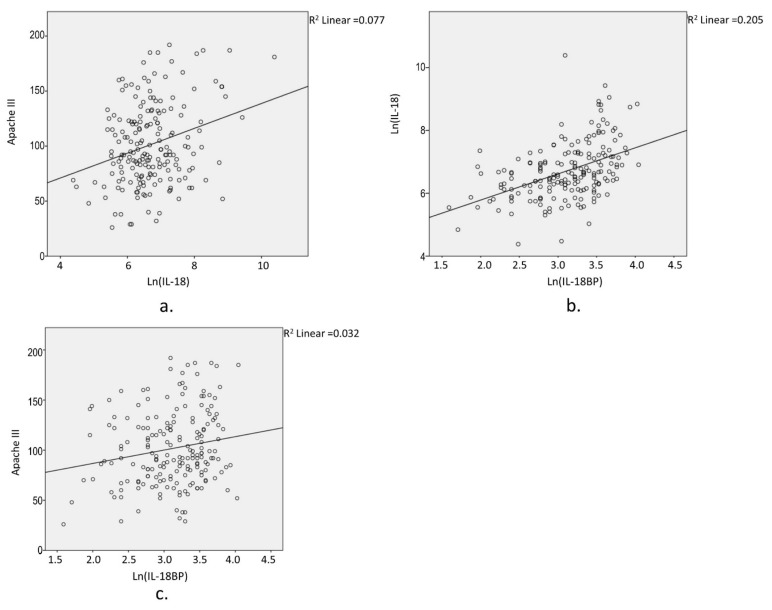
Correlations of Ln (IL-18), Ln (IL-18BP), and Apache III score in bacterial sepsis. The plots presented demonstrate the moderating effects of IL-18BP. To assess the correlation between IL-18 and Apache III score (**a**), the correlation between IL-18BP and IL-18 (**b**), as well as the potential correlation between IL-18BP and Apache III score (**c**), was controlled for in this study.

We observed a wide dynamic range of IL-18 plasma concentrations, from 80 to >32,400 pg/mL in bacterial sepsis, and from 161 to 19,100 pg/mL in viral sepsis. Compared to plasma LIGHT levels, the increase in plasma IL-18 levels showed only nominal significance in patients with sepsis (Table 2). In our study, sex, age, and BMI did not show a correlation with IL-18 levels. However, IL-18 levels were found to correlate with self-identified race, with African American subjects manifesting lower IL-18 levels than other populations (6.44 ± 0.98 vs. 6.79 ± 0.85, *p* = 0.003). In this case, instead of defining abnormal IL-18 levels, we tested the association of quantitative IL-18 levels with clinical phenotypes, controlled for race (Table 3). Overall, IL-18 levels were correlated with Apache III scores, mortality, and AKI, with highly significant *p*-values, and correlated with AHRF and ARDS with nominal significance. In addition, the correlation of IL-18 with Apache III scores, mortality, and AKI was consistently observed in both bacterial sepsis and viral sepsis. In contrast, the correlation of IL-18 with AHRF was only observed in viral sepsis. These results suggest that, unlike LIGHT with prominent effects mainly observed in bacterial sepsis, IL-18 has a significant impact in both bacterial and viral sepsis. Furthermore, considering that IL-18 can be neutralized by IL-18BP (Figure 1), we also tested the IL-18 effects corrected for IL-18BP (Table 3).

### 3.4. Other Biomarkers in Sepsis

The 59 exploratory biomarkers include a comprehensive list of inflammatory analytes and pathways integrated in the Human Inflammation MAP^®^ v. 1.1 multiplexed immunoassays. Among the 59 exploratory biomarkers tested (i.e., markers other than LIGHT and IL-18 (Table S3)), the most significant associations were as follows tissue inhibitor of metalloproteinases 1 (TIMP-1) was positively correlated with Apache III, with highly significant *p*-values in both bacterial and viral sepsis; plasminogen activator inhibitor 1 (PAI-1) was positively correlated with ARDS and AHRF in both bacterial and viral sepsis; tumor necrosis factor receptor 2 (TNFR2) was positively correlated with AKI in both bacterial and viral sepsis (Table 4). In bacterial sepsis, LIGHT was positively correlated with TIMP-1, PAI-1, and TNFR2. The above associations of LIGHT with these disease-associated biomarkers are in keeping with the effects of LIGHT that we observed in association with severe bacterial sepsis and its complications. IL-18 levels were correlated with disease biomarkers in both bacterial and viral sepsis. IL-18 levels were not correlated with elevated LIGHT levels in either bacterial or viral sepsis.

## 4. Discussion

### 4.1. LIGHT and IL-18 Levels as Biomarkers in Sepsis

In this study, we report elevated LIGHT levels in both bacterial and viral sepsis. In bacterial sepsis, LIGHT is correlated with extended length of hospitalization, increased sepsis severity, and sepsis complications. Viral sepsis cases had higher prevalence of ARDS and AHRF than bacterial sepsis. The lack of correlation of LIGHT in viral sepsis with Apache III score, ARDS, AHRF, and AKI is likely a reflection of the smaller sample size (*n* = 91).

In contrast to LIGHT as a mediator of immunoregulation, IL-18 is a proinflammatory cytokine, inducing the production of interferon-γ (IFNγ) [36]. A previous study suggested IL-18 as a potential therapeutic target due to its crucial roles in sepsis [7]. In our study, IL-18 levels were consistently observed for correlations with increased sepsis severity and sepsis complications in both bacterial and viral sepsis. IL-18BP is the specific inhibitor of IL-18 and neutralizes IL-18 activities [36]. A previous study showed that IL-18 mediates ischemic acute tubular necrosis in AKI [37], whereas our study shows that IL-18BP has much stronger correlation with AKI than IL-18 in sepsis. Except for AKI, the IL-18 effects remained significant after correction for IL-18BP. In our study, IL-18 levels were not correlated with elevated LIGHT levels in either bacterial or viral sepsis, suggesting independent effects of LIGHT and IL-18 in sepsis, rendering a combination therapy with neutralizing antibodies to LIGHT and IL18 a feasible choice.

### 4.2. LIGHT/IL-18 Levels and Apache III Score

The association of increased LIGHT level and Apache III score in bacterial sepsis, as well as the highly significant correlation of IL-18 with Apache III score in both bacterial and viral sepsis, observed in this study may be explained in part by the association of LIGHT/IL-18 with increased risk of ARDS, AHRF, and AKI.

TIMP-1 is the most significant biomarker of Apache III score in both bacterial and viral sepsis, as shown in this study. TIMP-1 is a key regulator of degradation of extracellular matrix. The matrix metalloproteinases (MMPs) degrade extracellular matrix and modulate cytokine and chemokine activity, which play important roles in immune responses to infection [38]. TIMP-1 is a natural inhibitor of MMPs [39]. TIMP-1 also functions as a cytokine, promotes cell proliferation, and has antiapoptotic function [40,41]. The study by Lorente et al. showed increased level of TIMP-1 as a biomarker to predict the clinical outcome of patients with sepsis [42].

Significantly increased expression of TIMP-1 by activated leukocytes has been observed in inflammatory tissues [43,44]. As shown by our study, LIGHT is positively associated with TIMP-1 in bacterial sepsis, which may be related to its roles in the function of activated T cells [17,18,19] and systemic inflammatory activation [20]. IL-18 is consistently correlated with TIMP-1 with highly statistical significance in both bacterial and viral sepsis (Table 4). We observed a positive correlation between IL-18 and IL-6/IL-10 in both bacterial and viral sepsis (Table S3). Previous studies have shown that IL-6 induces the synthesis of TIMP-1 [45], and that IL-10 enhances release of TIMP-1 from peripheral blood mononuclear cells (PBMCs) [46].

### 4.3. LIGHT/IL-18 Levels and ARDS/AHRF

PAI-1 levels show a significant correlation with ARDS and AHRF, which are also positively correlated with LIGHT levels in bacterial sepsis and positively correlated with IL-18 levels in both bacterial and viral sepsis. PAI-1 is the main physiological plasminogen activator inhibitor, which is critical for regulating the fibrinolytic system and maintaining normal hemostasis [47]. Disseminated intravascular coagulation (DIC) is an important pathogenesis in the early stage of acute lung injury (ALI) and ARDS [48]. Due to the crucial role of the fibrinolytic system and DIC in the pathophysiology of sepsis, PAI-1 levels have been shown to be important prognostic biomarkers in sepsis [49]. Elevated in lung injury, alveolar PAI-1 levels can also predict ARDS in aspiration pneumonitis [50]. Encoded by the serpin family E member 1 gene (*SERPINE1*), the expression of PAI-1 is upregulated by LIGHT [51]. Therefore, the correction between LIGHT/IL-18 levels and PAI-1 levels identified by this study suggests that LIGHT/IL-18 may contribute to ARDS/AHRF in sepsis by increasing the PAI-1 levels.

### 4.4. LIGHT/IL-18 Levels and AKI

In our study, TNFR2 was significantly correlated with AKI in both bacterial and viral sepsis. TNFR2 is the second receptor of the cytokines TNF and lymphotoxin-α, shown to mediate both proinflammatory and anti-inflammatory effects [52]. In recent years, TNFR2 has attracted research attention as an emerging drug target for autoimmune diseases and cancer [52]. LIGHT levels in bacterial sepsis and IL-18 levels in both bacterial and viral sepsis were positively correlated with TNFR2. Both LIGHT and TNFR2 are integral components of the lymphotoxin system [17]. TNFR2 is encoded by the TNF receptor superfamily member 1B gene (*TNFRSF1B*) with restricted expression, e.g., in endothelial cells (ECs) [53] and lymphoid cells [54]. In the immune system, TNFR2 is predominantly expressed by a subset of highly suppressive CD4^+^CD25^+^FoxP3^+^ regulatory T cells (Treg) upon T-cell receptor (TCR) activation [55,56]. The correlation of LIGHT with increased TNFR2 levels may be due to the supportive effects of LIGHT on the expansion and function of Tregs [57], while IL-18 has been shown to drive Treg differentiation [58].

In sepsis, TNFR2 has been shown to associate with CD4^+^ T-cell impairment and post-septic immunosuppression by activation of Tregs [59]. In addition to immunosuppression, significant upregulation of TNFR2 in kidney injury was observed on tubular epithelial cells, including both distal convoluted tubules and proximal convoluted tubules [60]. Renal-expressed TNFR2 promotes renal monocyte recruitment by the IRF1 and IFN-β autocrine signaling [53], which may contribute to renal injury in sepsis. LIGHT expression by mucosal T cells has been shown to promote TNFR2 expression in inflammatory tissue [61]. As the interferon-γ-inducing factor, IL-18 may increase the expression of TNFR2 in kidney through interferon-γ, and it has been shown to stimulate renal epithelial cells to express TNFR2 [62].

## 5. Conclusions

This study demonstrated independent associations of LIGHT and IL-18 in septic organ failure. Our study highlighted two potential therapeutic targets in sepsis, i.e., the significantly increased LIGHT levels and the highly variable IL-18 levels across a subset of patients with sepsis. The detrimental roles of LIGHT in multi-organ failure were mainly seen in bacterial sepsis, including ARDS/AHRF, AKI, and its association with higher Apache III score, as well as length of hospital stay, with a significant trend toward higher mortality rates. Consistently, the detrimental effects of LIGHT in bacterial sepsis were supported by the correlations of LIGHT with other biomarkers of organ failure, suggesting a key role for LIGHT as an inflammatory driver of other detrimental mediators [63]. Given that LIGHT presents an interesting therapeutic target for severe inflammatory conditions, our results suggest for the first time that anti-LIGHT therapy with neutralizing mAbs may be effective in a subset of patients with sepsis unrelated to COVID-19 infections. In contrast, IL-18 was associated with the detrimental outcome of both bacterial and viral sepsis, including increased mortality, and additionally supported by the correlations of IL-18 with other biomarkers involved with organ failure. The observed significant variance in IL-18 levels between individuals with sepsis highlights its potential as a precision therapeutic target with neutralizing mAbs, by selecting patients with significantly elevated IL-18. The lack of correlation between LIGHT and IL-18 levels, as well as different correlations with other biomarkers, suggests independent and distinct roles of LIGHT and IL-18 in sepsis, and that therapy directed against both of these cytokines should be given consideration.

## Figures and Tables

**Table 1:** Clinical characteristics of the research subjects.

Clinical Information	Bacterial Sepsis (*n* = 189)	Viral Sepsis (*n* = 91)
**Age (median, 1st and 3rd quartiles)**	61.5 (52.1, 71.4)	61 (52.5, 69)
**sex**	111 (58.7%) males; 78 (41.3%) females	51 (56.0%) males; 40 (44.0%) females
**BMI ($\bar{x}$ ± s)**	27.05 ± 7.70	27.59 ± 7.37
**race**	White: 121; Black: 57; Asian: 8; Native American: 1; Other: 2	White: 55; Black: 31; Asian: 2; Other: 3
**ARDS**	Yes: 65 (34.4%); No: 124 (65.6%)	Yes: 36 (39.6%); No: 55 (60.4%)
**AHRF**	Yes: 71 (37.8%); No: 117 (62.2%)	Yes: 50 (55.6%); No: 40 (44.4%)
**AKI**	Yes: 114 (61.6%); No: 71 (38.4%)	Yes: 49 (53.8%); No: 42 (46.2%)
**APACHE III**	101.6 ± 36.9	90.8 ± 39.4
**LOS**	19.38 ± 20.78	17.70 ± 19.98
**Mortality**	Yes: 77 (40.7%); No: 112 (59.3%)	Yes: 33 (36.3%); No: 58 (63.7%)

**Table 2:** Ln (LIGHT) and Ln (IL-18) levels in bacterial and viral sepsis.

Group	*n*	Ln(LIGHT) ^#^		Ln(IL-18) ^#^	
		$\bar{x}$ ± s	*p*	$\bar{x}$ ± s	*p*
**Bacterial sepsis**	189	4.46 ± 0.71	1.80 × 10^−5^	6.71 ± 0.91	0.038
**reference controls**	22	3.76 ± 0.64		6.28 ± 0.87	
**Viral sepsis**	91	4.29 ± 0.72	1.78 × 10^−3^	6.63 ± 0.90	0.048
**reference controls**	22	3.74 ± 0.70		6.20 ± 0.89	
**Total**	280	4.40 ± 0.72	3.46 × 10^−8^	6.68 ± 0.90	2.80 × 10^−3^
**reference controls**	44	3.75 ± 0.66		6.24 ± 0.87	

^#^ LIGHT and IL-18 levels are in pg/mL.

**Table 3:** Correlation of quantitative Ln (IL-18) levels with organ failure.

IL-18^#^	All Cases		Bacterial Sepsis		Viral Sepsis	
	***r***	***p*-value**	***r***	***p*-value**	***r***	***p*-value**
**ARDS**	0.121 *	0.045	0.103	0.165	0.167	0.117
**AHRF**	0.145 *	0.017	0.101	0.175	0.251 *	0.018
**AKI**	0.187 **	0.002	0.151 *	0.041	0.257 *	0.015
**Apache III**	0.301 **	0.000	0.278 **	1.40 × 10^−4^	0.340 **	0.001
**LOS**	0.034	0.577	0.064	0.392	−0.035	0.743
**Mortality**	0.288 **	0.000	0.254 **	0.001	0.350 **	0.001
Corrected for IL-18BP by partial correlation^#^	***r***	***p*-value**	***r***	***p*-value**	***r***	***p*-value**
**ARDS**	0.144 *	0.018	0.147 *	0.047	0.16	0.135
**AHRF**	0.163 **	0.007	0.15 *	0.043	0.228 *	0.033
**AKI**	0.091	0.135	0.084	0.262	0.127	0.24
**Apache III**	0.239 **	7.08 × 10^−5^	0.223 **	0.003	0.272*	0.01
**LOS**	0.085	0.162	0.142	0.056	−0.023	0.829
**Mortality**	0.269 **	7.34 × 10^−6^	0.242 **	0.001	0.321 **	0.002

^#^ Controlled for race. * *p* < 0.05; ** *p* < 0.01.

**Table 4:** Correlation of elevated LIGHT and Ln (IL-18) with biomarkers of organ failure.

	apacheIII		ARDS		AHRF		AKI		LIGHT		IL-18	
**Bacterial sepsis**	***r***	***p*-value**	***r***	***p*-value**	***r***	***p*-value**	***r***	***p*-value**	***r***	***p*-value**	***r***	***p*-value**
Interleukin-18 (IL-18)	**0.277** **	**1.13 × 10^−4^**	0.077	0.295	0.076	0.298	**0.145** *	**0.049**	0.130	0.074	1	
Interleukin-18-binding protein (IL-18bp)	**0.179** *	**0.014**	−0.060	0.413	−0.073	0.319	**0.174** *	**0.018**	**0.178** *	**0.014**	**0.453** **	**6.19× 10^−11^**
Plasminogen activator inhibitor 1 (PAI-1)	**0.287** **	**6.32 × 10^−5^**	**0.215** **	**0.003**	**0.223** **	**0.002**	**0.274** **	**1.57 × 10^−4^**	**0.210** **	**0.004**	**0.264** **	**2.36 × 10^−4^**
Tissue inhibitor of metalloproteinases 1 (TIMP-1)	**0.372** **	**1.35 × 10^−7^**	0.111	0.130	0.112	0.125	**0.279** **	**1.21 × 10^−4^**	**0.224** **	**0.002**	**0.414** **	**3.11 × 10^−9^**
Tumor necrosis factor receptor 2 (TNFR2)	**0.353** **	**6.12 × 10^−7^**	0.076	0.296	0.045	0.537	**0.332** **	**3.89 × 10^−6^**	**0.225** **	**0.002**	**0.537** **	**1.74× 10^−15^**
**Viral sepsis**	**r**	***p*-value**	***r***	***p*-value**	***r***	***p*-value**	***r***	***p*-value**	***r***	***p*-value**	***r***	***p*-value**
Interleukin-18 (IL-18)	**0.361** **	**4.43 × 10^−4^**	0.123	0.247	0.203	0.054	**0.234** *	**0.026**	−015	0.885	1	
Interleukin-18-binding protein (IL-18bp)	**0.266** *	**0.011**	0.034	0.746	0.088	0.408	**0.381** **	**1.92 × 10^−4^**	−0.070	0.512	**0.408** **	**6.01 × 10^−5^**
Plasminogen activator inhibitor 1 (PAI-1)	**0.416** **	**4.12 × 10^−5^**	**0.235** *	**0.025**	**0.263** *	**0.012**	**0.248** *	**0.018**	0.056	0.596	**0.379** **	**2.08 × 10^−4^**
Tissue inhibitor of metalloproteinases 1 (TIMP-1)	**0.554** **	**1.21 × 10^−8^**	**0.277** **	**0.008**	**0.303** **	**0.004**	**0.368** **	**3.29 × 10^−4^**	−0.079	0.455	**0.560** **	**8.12 × 10^−9^**
Tumor necrosis factor receptor 2 (TNFR2)	**0.467** **	**3.11 × 10^−6^**	0.124	0.242	0.178	0.094	**0.363** **	**4.06 × 10^−4^**	−0.033	0.754	**0.594** **	**5.47 × 10^−10^**

* *p* < 0.05; ** *p* < 0.01.

## Data Availability

Supporting data from this study can be obtained by emailing the corresponding author Hakon Hakonarson.

## Supplementary Materials

The following supporting information can be downloaded at: https://www.mdpi.com/article/10.3390/biomedicines10020264/s1: Table S1. Sixty-one Inflammation biomarkers investigated in this study; Table S2. Scanning for LIGHT cutoff by association tests; Table S3. Correlation of elevated LIGHT and Ln (IL-18) with biomarkers of organ failure.
